# The ethical and validity conundrum in epilepsy research in LMIC settings

**DOI:** 10.3389/fneur.2023.1196261

**Published:** 2023-05-17

**Authors:** Pauline Samia, Adeel Shah, Archana Patel, Philip Olielo, Lionel Mudave, Samson Gwer

**Affiliations:** ^1^Department of Paediatrics and Child Health, Medical College, Aga Khan University, Nairobi, Kenya; ^2^Brain and Mind Institute, Aga Khan University, Nairobi, Kenya; ^3^Department of Public Health and Primary Care, Ghent University, Ghent, Belgium; ^4^Division of Epilepsy and Clinical Neurophysiology, Department of Neurology, Boston Children's Hospital and Harvard Medical School, Boston, MA, United States; ^5^School of Medicine, Kenyatta University, Nairobi, Kenya; ^6^Department of Neurology, Gertrude's Children's Hospital, Nairobi, Kenya; ^7^Afya Research Africa, Nairobi, Kenya

**Keywords:** ethics, sustainable collaborations, epilepsy, low-middle-income-countries, research

## Introduction

In the last few decades, research in epilepsy has significantly improved understanding of risk factors and etiologies associated with epilepsy, promoting greater access to interventions and medications that have improved health-related outcomes for patients. However, these advances and benefits are not being felt evenly on a global scale due to significant inequalities in access to and utilization of research resources and expertise in Low-and Middle-Income Countries (LMICs).

To promote effective research output, and advance evidence-based practices; the context, disease burden, and challenges that hinder good research need to be re-defined and addressed. This is key in facilitating implementation of coherent priorities and strategies in epilepsy research in LMICs; and in facilitating the conduct of scientifically and ethically valid research. This paper explores the capacity, ecosystem, and ethical issues that are at play and that need to be addressed to support better evidence generation and utilization in epilepsy care in LMICs.

## Heterogeneity of settings in LMICs

The epidemiological profile of diseases and the health system landscape varies across regions in LMICs. For instance, HIV, malaria, onchocerciasis, cysticercosis, and sickle cell disease significantly alter the burden, presentation and outcomes of epilepsy (1–4). These diseases have an endemicity and prevalence profile that varies significantly across regions in LMICs and their impact on presentation of epilepsy in these regions is not even. Access to healthcare also varies across regions, as do socio-cultural practices. Poor access to care is associated with dismal maternal and newborn health outcomes, and by extension, the significant related neurological conditions including epilepsy. Such disparate access and outcome indicators are apparent between urban and rural settings and alter the presentation, management, and outcomes of epilepsy in these regions (2, 3). These differences are apparent in epilepsy neuro-imaging studies in rural Kenya and Tanzania that demonstrate infectious diseases as the predominant findings in contrast to similar studies in urban Kenya and South Africa, where imaging findings related to infectious etiologies are little observed (4). Metabolic diseases are much more prominent in epilepsy studies in North Africa compared to those in sub-Saharan Africa while the preference for traditional healers as the first contact in health-seeking behavior may be more common in Sub-Saharan Africa compared to other parts of Africa (4, 5).

There are few epilepsy studies in LMICs which are often concentrated in specific settings likely because of established capacity and research relationships. Because of the paucity of data, these studies are considered universally representative of LMIC settings. Therefore, there needs to be a recognition that the generalizability of findings in LMIC epilepsy research and the definition of priorities for intervention is limited and needs to take into account the heterogeneity of settings in LMICs (6).

## Support for researchers and drivers of the epilepsy research agenda in LMICs

Africa hosts 15% of the world's population but contributes 1.3% of global health research publications (7). Building and sustaining epilepsy research capacity in Africa and other LMICs is critical in addressing the disparities in epilepsy care. However, the epilepsy research capacity in most LMICs is limited. This is on account of gaps in resources, institutional base, research infrastructure, relevant training programmes, career development pathways, and regulatory environment and networks (8). In the context of limited resources, epilepsy is little prioritized in LMICs research agenda in favor of infectious diseases.

Most epilepsy research funding in LMICs is from external actors. Such funding usually comes with a predefined research agenda and anticipates competitive proposals in languages that are not native to the target countries. The funding calls usually expect collaboration with researchers from high income countries (HIC). However, the gaps in research capacity means that native LMIC researchers are usually not able to take the lead in such opportunities (2, 9). Thus, in many instances, the research projects are defined and led by collaborating HIC partners. This results in a high risk for biased perspectives from HIC partners, with uneven collaborative research relationships that limit ownership and recognition of the research process and output by LMIC partners and negatively impact the validity of such research efforts (10, 11). The adverse effects of such lopsided relationships has been brought to the fore in recent legal cases (12, 13). Researchers in LMICs would have a greater awareness of the epidemiology of epilepsy in their own settings. The future of sustainable global HIC and LMIC research relationships lies in equitable participation, recognition and attribution in the process and final outcomes, as well as in equipping young researchers with requisite skills allowing them to compete effectively for available resources and contribute to research projects at high standards (1, 10, 11, 14–17).

In 2022, the world health assembly adopted the World Health Organization (WHO) Intersectoral Global Action Plan on Epilepsy and other Neurological Disorders (IGAP), a strategy that prioritizes epilepsy and other diseases that negatively impact brain health globally. This plan will guide countries on the implementation of policies that lead to a reduction of the burden related to epilepsy and other neurological conditions (18, 19). The fourth objective of this strategy specifically targets the strengthening of research and information systems as well as the implementation of technology which are key to the improvement of outcomes for patients with epilepsy (18, 19). The definition of such global policies and strategies in epilepsy research and care is important in aligning the research agenda for various stakeholders, including funders, governments and researchers.

Alongside such aligned agenda definitions is the need to enhance intellectual property protection. Patents and copyrights are a vital part of medical research. They offer the researcher the opportunity to claim ownership and attribution of research output and obtain earnings where applicable. Patenting and copyright law enforcement are weak in LMICs, particularly in Sub-Saharan Africa (20, 21). Few countries have actively implemented the recommended Intellectual property protection laws. Cameroon has the Organization Africaine de la Propriete Intellectuelle (OAPI) and South Africa has the African Regional Intellectual Property Organization (ARIPO), organizations which implement deliberate government effort to promote data protection for their researchers. These agencies are relevant to LMICs aspirations to advance research in epilepsy and other non-communicable diseases (20, 21).

## Data systems, data protection and subject autonomy in epilepsy research in LMICs

Good data systems are essential for research. Most LMICs have poor health data systems and little capability to plan and learn from the data they collect. Health records and data systems are still paper-based and disjointed, making for limited ability to synthesize the data to guide practice and research. In many instances, epilepsy and other NCDs are not included in reporting systems. The result is that whole health systems are blind to epilepsy and other NCDs, and do not include them in resource planning or defining research agendas. In these settings, data protection systems are weak. This means that often, personal data is shared without the necessary ethical safeguards and respect for privacy. Rules and regulations around data protection are key for enhanced practices in research (22–25). This is particularly important considering that epilepsy study subjects in LMICs are uniquely more vulnerable than in other settings (26, 27). Quite often, they have limited access to care and their participation in research studies is their only opportunity for access (26, 27). They have less education and awareness and are often victims of stigma and disabling myths that impair their understanding of the importance of their participation in research. This can be muddled further by co-morbid cognitive and learning difficulties, and the loss of autonomy within the family and community setup. Their understanding of data protection issues can be limited. On the other hand, research is necessary to provide a greater understanding of their diseases and to promote optimal care for better outcomes (26, 27). In conducting research on subjects with epilepsy in LMICs, it is important to be deliberate in defining measures for greater individual protection, clear communication, direct benefits, and effective stakeholder participation (26). Data protection safeguards include the establishment of norms and mechanisms for monitoring practices, as well as promoting participant awareness of rights. Researchers have obligations to ensure they are meeting the ethical requirements and maintaining good clinical practices as relevant in the region where the research is conducted, as well as for all partnered ethical reviews. This can be a financial and time burden that warrants special consideration in funding for LMICs where such systems are often underdeveloped (25–29).

## Way forward

Communicable diseases that contribute to the occurrence of epilepsy have been extensively studied in LMIC settings (1). This focus needs to be reviewed in the context of improving survival and the increasing burden of non-communicable diseases. Given the varied etiology of epilepsy in LMICs, a targeted approach is needed when conducting research in these settings. Research priorities should be determined by the individual LMIC countries to ensure that it is not only relevant to them but also helps to address existing gaps in healthcare delivery. Due to existing disparities within individual countries, it is necessary to develop a comprehensive and strategic approach when conducting research, especially in areas of public health and implementation science. It is also important to target primary health care levels, to explore solutions that are devolved to where the burden is greatest. It is also essential that persons with epilepsy, their families, and local organizations are included in the research development and dissemination. Through participatory action and adherence to the principles of beneficence, research objectives should bear direct relevance for patients and increase the likelihood of impactful knowledge generation and interventions. This approach increases the likelihood of acceptance and implementation of the research outcomes.

In order to ensure local ownership and greater participation in epilepsy research there needs to be protected, equitable and sustained support for local researchers and healthcare providers who not only conduct the research but also provide most of the frontline care to persons living with epilepsy. Avenues for this support are not limited to research grants alone should also include improving access to information, training, and mentorship, and creating the necessary infrastructure and conditions to sustain and grow research in the LMICs. It is important to support education of trainee healthcare workers so that they understand the nuances involved in relevant epilepsy research planning, funding processes, the contribution of power relationships and how to manage them and how to generate data relevant to advocacy for further epilepsy research that is context specific.

**Figure 1:**
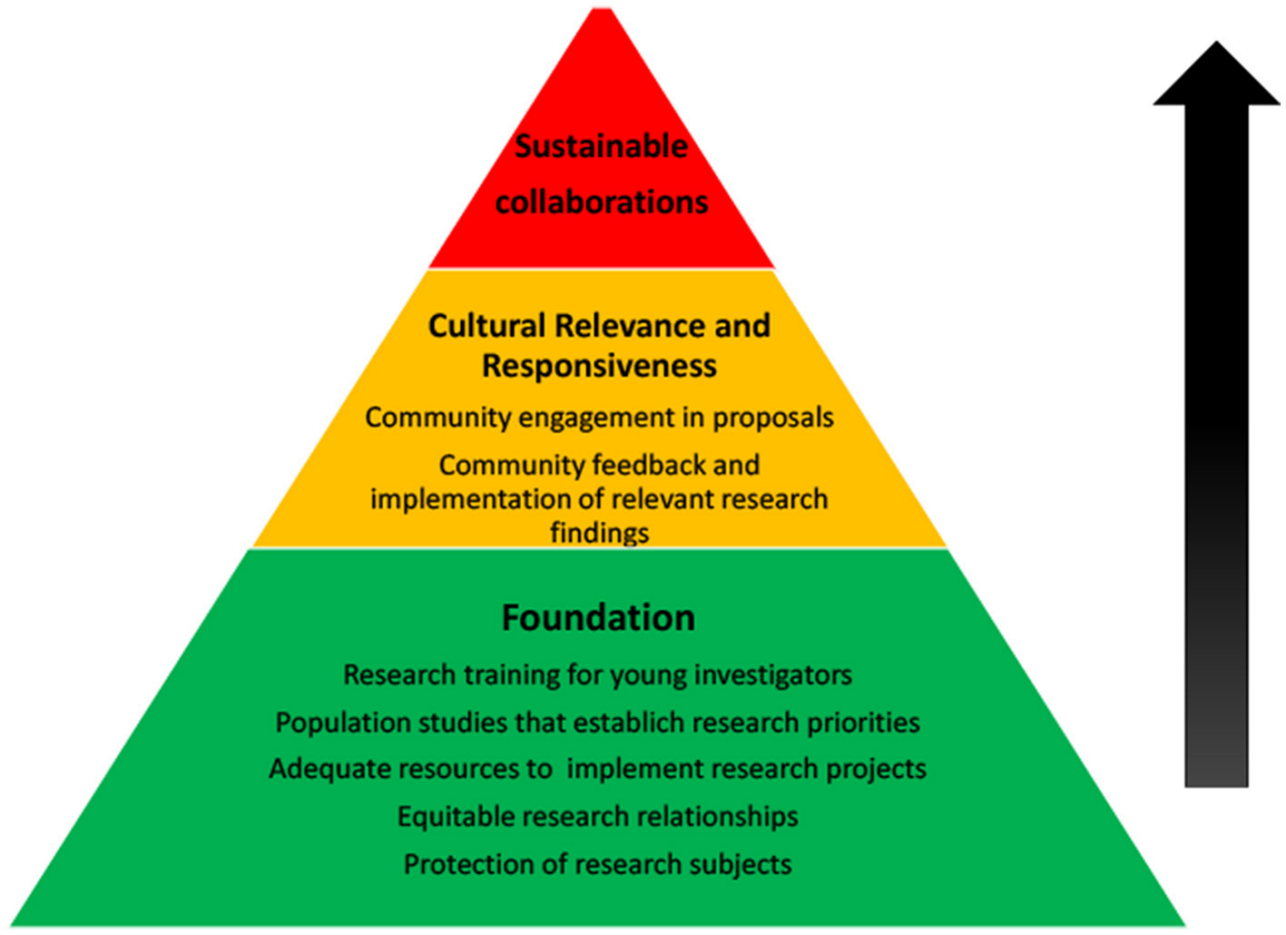
Ethical and sustainable research collaborations. This continuum pyramid above represents three levels of potential collaborative effort. These levels include sequential strategies and activities that contribute toward the attainment of sustainable epilepsy research collaborations.

Ethics review committees need to be aware of epilepsy comorbidities such as intellectual disability and depression and should require greater rigor during consenting process. The should require significantly simplified consent forms to enhance full understanding. Opt out options from the research process should be clearly outlined and the principle of autonomy emphasized to participants with epilepsy. It may be necessary to define a universal database for epilepsy observational studies as is the practice with clinical trials and systematic reviews, supporting greater accountability for ethical practices in epilepsy research. Such a platform could be managed and controlled by the international league against epilepsy (ILAE) and the international bureau for epilepsy (IBE), bodies that have global memberships and presence and are already invested in various aspects of epilepsy work. An implementation toolkit and the definition of generic policy and strategy documents would help the implementation of IGAP principles which attempt to align various stakeholders in the fight against epilepsy and other neurological diseases. Figure 1 summarizes the main requirements for equitable and sustainable global research collaborations.

In conclusion this paper advocates for the close alignment of research priorities with the real needs of the people living with epilepsy and with participation of local researchers in LMIC settings. The recognition of the heterogeneity of disease epidemiology and socio-cultural practices; implementation of best practices in research collaborations; development of research capacity and infrastructure; and adherence to ethical principles in the context of vulnerable subjects and weak systems; are key elements for in promoting better epilepsy research and care in LMICs. It is imperative that these factors inform the epilepsy research focus in LMIC settings where resources are scarce and the return on invested effort needs to have the widest impact achievable.

## Author contributions

PS, AS, AP, and SG contributed to the conceptualization of the paper. AS, PO, and LM reviewed literature relevant to the publication. PS, AS, LM, PO, AP, and SG contributed to the write-up and review of the manuscript and approved the final submission.
